# Repetitive Rolling of Triptycene-Based Molecules on Cu Surfaces

**DOI:** 10.1021/jacs.4c08652

**Published:** 2024-09-19

**Authors:** Mine Konuk, Melihat Madran, Mehmet Tuna Uysal, Deniz Beşer, Alimet Sema Özen, Zehra Akdeniz, Sondan Durukanoğlu

**Affiliations:** † Faculty of Engineering and Natural Sciences, Kadir Has University, Istanbul 34083, Türkiye; ‡ Faculty of Engineering and Natural Sciences, Sabancı University, Istanbul 34956, Türkiye; § Robert College, Istanbul 34345, Türkiye; ∥ Faculty of Arts and Sciences, Piri Reis University, Istanbul 34940, Türkiye

## Abstract

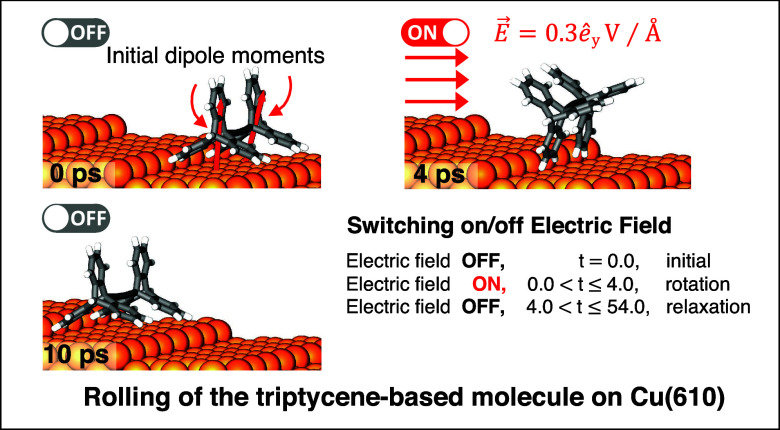

The metal surface-supported rotation of artificial molecular structures is technologically important for developing molecular-level devices. The key factors leading to the practical applications of these molecular machines on metal surfaces are the atomic-scale control of the rotation and the counterbalance of the temperature-driven instability of the molecules. In this work, we present a means by which triptycene-based molecular wheels can roll repetitively on a metal surface. Our results show that regularly stepped surfaces are the perfect candidate not only for stabilizing the molecule on the metal surface but also for providing the pivot points needed for repetitive vertical rotation of the molecule at higher temperatures. In addition to the geometrical compatibility of the substrate and the molecule, intermittent application of the external electric field is needed for rolling the molecule on a metal-stepped surface in a controllable manner.

## Introduction

Molecular nanotechnology has reached such an advanced level that it can facilitate the design, synthesis, and control of single-molecule machines with precision at the atomic scale.^1−3^ In addition, manipulation of the motion and performance of these molecules can be achieved at a molecular level.^4^ Although various driving mechanisms for the rotation of a molecule have been introduced,^5−8^ there have been a few reports on the dynamics and control of a repetitive vertical motion of a molecule on a metal substrate.^9−11^ For instance, Rappenne and his co-workers synthesized a nano wheel with a simple geometry formed by two triptycene groups connected by a carbon chain.^12,13^ Furthermore, by pushing the molecule on a rough surface of Cu(110) with a scanning tunneling microscopy (STM) tip, they were able to roll and slide the molecule for the first time.^14^ However, STM manipulations were performed at very low temperatures and repetitive rolling of the molecule on the surface was not achievable.

Isolated single molecules may have vanishing dipole moments. When placed on a metal surface, the charge transfer between the molecule and the surface induces a dipole moment strong enough to interact with the external electric field (EF).^11,15−18^ The torque resulting from this interaction may cause the vertical rotation of the molecule on the substrate, provided the surface geometry is rough enough to accommodate a pivot point.^11,19^ The roughness of the surface is crucial to increase the torque on the molecule and counterbalance the thermally induced destabilization of the molecule on the surface.^8^ Thus, when the ultimate goal is to have a repetitive rolling of a molecule on a metal substrate, applying a finely tuned external electric field (EF) and geometrically tuned surface roughness might be a natural alternative.

To this end, we conducted extensive calculations on the molecular rotation of triptycene-based molecular wheels induced by a metal tip and an external EF on the Cu(110) flat and various vicinal surfaces. Among the vicinal surfaces with terraces wide enough to allow the molecule to rotate without sliding, we specifically focused on the vicinal surface of Cu(610). This surface offers perfectly spaced steps with more openly arranged atoms, making it possible for the molecule to firmly attach and subsequently rotate without any sliding. We found that the repetitive rotation of the molecular wheels can be achievable under two conditions: first, the surface geometry of the substrate is compatible with the rotating wheels of the molecule; second, the external EF is applied intermittently so that the molecule and pivot points of the repetitive rotation can be stabilized. Our simulations further suggest that vicinal surfaces are perfect candidates for controlling the random thermal motion of the molecule on metal surfaces.

## Computational Details

### Geometry

**Figure 1:**
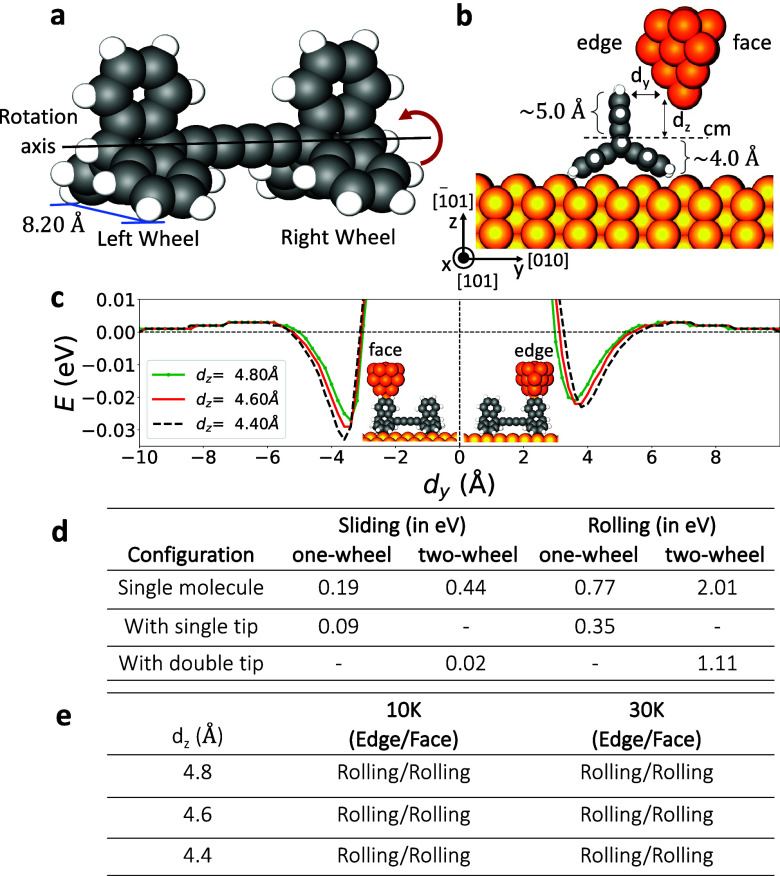
(a) Representation of triptycene-based molecular wheels: gray and white colors show carbon and hydrogen atoms, respectively. (b) Position of the Cu-tip with respect to the surface and the molecule before the manipulation. (c) Potential energy landscape of the tip with respect to the molecule. Inset indicates the types of Cu-tip. (d) Activation energy barriers (in eV) to slide/rotate one wheel and two wheels of the triptycene-based molecule on the Cu(110) surface. (e) MD results for the movement of the molecule with a single Cu-tip at temperatures of 10 K, 30 K. *d*_*z*_ shows the height of the Cu-tip with respect to the center of mass of the molecule; edge and face refer to types of Cu-tips. The color codes in the figure are orange for Cu atoms, dark orange for Cu-tip atoms, gray for carbon atoms, and white for hydrogen atoms.

The computational cells for investigating the rotation of the triptycene-based molecular wheels contain a Cu substrate, a two-wheel molecule, and a Cu metal tip when needed. The two-wheel molecule is made up of 44 carbon atoms and 26 hydrogen atoms and is synthesized by connecting the two wheel-like triptycene molecules via a carbon axle (see Figure 1a). Two types of Cu substrates are utilized in the simulations: the flat surface and the vicinal surface. The computational cell details for all substrates are listed in Table S1. For instance, the slab for Cu(110) accommodates 22 × 14 × 12 atoms along the *x*-, *y*-, and *z*-directions, whereas the slab for Cu(610) consists of 8 × 42 × 10 atoms (see Figure S1a,b). No periodic boundary condition was implemented along the *z*-direction, whereas computational cells holding the two-wheel molecule were held periodic along the *x*- and *y*-directions. In this report, the height of the Cu tip, *d*_*z*_, was taken as the separation between Cu tip-apex and the center of mass of the molecule (see Figure 1b). A triangularly shaped Cu-cluster with sharp edges is designed to mimic the experimental tip, and the architecture provides two different tip orientations to manipulate the molecule: the edge and the flat face of the Cu-cluster (see Figure 1b and the insets in Figure 1c).

### MD Simulations

We employed the third generation of charge-optimized many-body (COMB3) potentials to characterize various interactions in the model systems, which captures the chemical and physical aspects of the distinct bond types.^20^ COMB3 potential is a charged-optimized empirical potential that has so far proven to accurately predict the formation and breakage of hydrocarbon bonds on Cu surfaces,^21^ wrinkle formation due to differences in thermal expansion coefficients between graphene and Cu substrates,^22^ and the early stages of copper surface oxidation.^20^ The charge state of an atom inside the COMB3 potential formalism can be dynamically calculated using the charge equilibration (QEq) method,^23^ in which the charge on each atom is set to minimize the system’s energy. All MD simulations have been executed using LAMMPS packages.^24,25^ The 0 K equilibrium arrangements of the atoms and the stable configuration of the molecule were determined by using the standard conjugate gradient method for total energy minimization. Nosé–Hoover thermostat was implemented to maintain systems in equilibrium at distinct temperatures. The velocity-Verlet algorithm was utilized to integrate the equations of motion of the systems with time steps of 0.2 fs. In the simulations the Cu slab with a single molecule was first minimized at 0 K, and then the resulting configuration was equilibrated for 100 ps at each elevated temperature. Cu-tip was separately minimized at 0 K and subsequently equilibrated with the system at the specified temperatures before any manipulation. After these equilibration steps, the velocity of the Cu tip was set to 0.5 Å/ps, and the molecule was manipulated by the tip within the duration of 50 ps. The temperature of the system, including Cu-tip was maintained at a set temperature throughout MD simulations. Activation energy barriers of the mechanisms were calculated using an accurate and efficient technique of the nudged elastic band (NEB).^26^ To execute NEB calculations for a complex transition such as the rolling mechanism, a well-converged transition path was needed. For that, at least 6 initial configurations of intermediate replicas were selected from MD trajectories to converge the NEB replica images correctly.

## Results and Discussion

### Structure and Energetics

Single molecules on surfaces can generally move via simple hopping, sliding, rolling, pivoting, or combinations of these mechanisms. Of the critical factors determining the type of motion is the orientational preference of the molecule with respect to the substrate. For triptycene-based molecular wheels, for example, it has been observed that although the molecule may adopt different orientations on the corrugated Cu(110) surface, it mainly prefers a specific arrangement in which the molecular axle is aligned with the closed-packed rows of the surface.^14^ This configuration is also attributed to the directional preferences of the wheels, which prompts the rolling of the molecule. Thus, we first constructed the potential energy surface (PES) of the molecule to determine its orientational preferences on the Cu(110) surface using constrained geometry optimization. To do that, we conducted total energy calculations based on COMB3 potentials to obtain the PES profiles for various heights in the course of 360° rotation of the molecule around the center of mass (see Figure S2). The outcomes of the PES profiles align with the experimental findings that the A, B, C, and D orientations are the most probable for the molecule to be positioned on the corrugated surface of Cu(110) (see Figure S2a,c). It is also worth noting that while the orientations A and B are energetically favorable regardless of the height of the molecule, the C and D orientations become less probable as the molecule is slightly separated from the substrate. As seen in Figure S2a, site A has a triggering effect on the rolling of the molecule with a Cu-tip because H atoms within the benzene rings are almost locked with the Cu atoms of the close-packed rows of the substrate, providing the pivot point for the wheel to rotate. Therefore, in our simulations, configuration A is considered the initial minimum energy position for manipulating the two-wheel molecule on Cu(110).

After determining the orientational preference and location of the molecule with respect to the surface, we run the simulations to differentiate the driving forces behind the tip-induced atomic manipulations. Since the molecule’s rotation on the surface is to be stimulated by mostly lateral forces, the changes in the lateral strength of the interaction between tip and molecule are expected to play a pivotal role. Therefore, we plotted the PES landscape of the tip with respect to the molecule as a function of lateral spacing in addition to vertical spacing in Figure 1c (for the lateral and vertical spacing see Figure 1b). As can be clearly seen from the PES landscape, varying vertical spacing reveals its effect, though small when the tip is in the close lateral vicinity of the molecule. In addition, the overall behavior of the PES graph is similar when the tip approaches the molecule with the face or edge orientation. As the tip approaches the molecule with the edge orientation, the potential energy reaches its minimum when the separation between the tip and the molecule is 3.8 Å. As expected, repulsive forces become very strong at smaller lateral distances (*d*_*y*_), as reflected by a sharp rise in the total energy. In contrast, attractive forces dominate for separations greater than the minimum energy spacing. The optimal range of 4.0–4.8 Å for the height of *d*_*z*_ of the tip, is carefully determined so that the molecule would experience the repulsive interaction needed for any type of motion, sliding or rolling while keeping the shape of the tip undeformed. Although *T* = 0 K energetic calculations may not be sufficient to fully understand molecular motors in their practical applications, they provide insight into the underlying physics of the collective interactions between the molecule, tip, and surface and the energy barriers the molecule has to overcome when it slides or rolls on the surface. To this end, we conducted total energy calculations to determine the activation energy barriers for different types of motions and reported the associated energy values in Figure 1d. From an energy point of view, sliding mechanisms for all configurations (with/without a single/double tip involving one/two-wheel motion) are energetically more favorable than rolling mechanisms as they cost less energy (see Figure 1d). While the presence of the tip reduces the energy barrier for the rolling process by half, it practically eradicates the barrier for the sliding mechanism, making the process almost readily achievable.

### Single-Tip-Induced Manipulation on Cu(110) at Various Temperatures

To understand the critical time- and temperature-dependent characteristics of the rotational motion of small molecules on metal surfaces, we also conducted MD simulations to observe specific types of motions of the two-wheel molecule on the Cu(110) surface when a Cu tip pushes it at higher temperatures. In the simulations, using the results of 0 K calculations, the initial position of a single Cu-tip is set to be 5 Å away from the one wheel of the molecule in the lateral dimension of *d*_*y*_, and then let move in the *y*-direction toward the molecule with a speed of 0.5 Å/ps for varying heights of the tip. The results are tabulated in Figure 1e. The interaction between the molecule and the tip stimulated the rotation of one wheel near the tip for each tip orientation (edge and face) at 10 and 30 K for vertical positions of the tip listed in the table. The other wheel moved away from its initial position in the process (see Movies S1 and S2). The molecule eventually ended up at configuration C shown in Figure S2a. Note that for all tabulated vertical positions of the tip, simulations at specific temperatures were repeated with the tip in both edge and face configurations, approaching the right and left wheels from both the back and front. The results listed in the table reflect the consistent outcomes of these repeated simulations. In contrast, as shown in Figure S3d,e, repeated simulations at 80 K for a specific tip height do not yield consistent characteristics in the motion of the molecular wheels. At this temperature, molecular wheels may exhibit sliding motion (see Movie S3), purely rotational motion, or a combination of both sliding and rotational motion on the corrugated Cu(110) surface (see Movie S4). This distinct nature of the motion of the wheels at elevated temperatures is understandable since the vertical spacing of the molecule with the substrate is governed by the thermal effects and the molecule may not experience the roughness of the (110) surface once it is thermally elevated.

In addition to a strong enough rotational force, pivot points of the rotation play a crucial factor in prompting the rotation of the molecule on a rough surface. Therefore, one must examine how the molecule is attached to a pivoting point during its rotational motion. One measure of the attachment of the two-wheel molecule on the Cu(110) surface is the distance between the H atoms of the wheels and the Cu surface. We, therefore, determined the relevant distances for varying temperatures and plotted them as a function of time steps during one rotational motion of the molecule stimulated by the Cu tip in Figure S3c,d,e. At 0 K, when there is no Cu tip, the initial altitudes of the H atoms at the pivoting point in the legs of the left and right wheels are not symmetric and found to be 1.74 and 1.77 Å, respectively. When the temperature was set to 10 K, the molecule kept its regular configuration with, this time, 1.67 and 1.88 Å altitudes of the H atoms of the corresponding wheels, respectively (see Figure S3a,c). When the temperature, on the other hand, was increased to 30 K, the respective positioning of the legs started to alter. The axial symmetry was broken at 80 K, leading to a disoriented orientation of the wheels (see Figure S3a). For both 10 and 30 K, the vertical separation plot indicates wide and deep basins, which may serve as a perfect pivoting point for rolling (see Figure S3c). However, at 80 K, the basin becomes shallower with a narrow basin compared to its 10 and 30 K counterparts due to the temperature-driven upward shift of the molecule from the surface. Therefore, the chances for the molecule to roll on the corrugated Cu(110) surface decrease significantly at high temperatures above 80 K. However, Grill et al. reported that the major factor determining the type of motion the molecule performs on the corrugated Cu(110) surface is the altitude of the tip,^14^ and the effects of temperature were not addressed. In the experiment, on the other hand, the STM images to observe the molecule’s motion were taken at temperatures between 20 and 30 K, which are quite low. Note that we also observed rolling of the molecule at low temperatures (10 and 30 K), but when the temperature is increased to 80 K or so, rolling becomes less probable because of the weakened interactions between the surface and the wheels of the molecule. In short, results from our simulations confirm the primary factors that cause the molecule to roll, the roughness of the surface and height of the tip, but introduce another important factor, temperature. In their experimental work, Grill and co-workers also reported the alternating rolling of two wheels, which was not observed in any of our simulations involving only one tip. To this end, we also introduced another tip for the second wheel to manipulate the molecule as a whole; instead of alternating rolling, a full rotation of the molecule is hardly observed at very low temperatures such as 10 K, regardless of the heights of the tip. All the details and the movies of the two Cu-tip systems on Cu(110) can be seen in Figure S4, Movies S5 and S6.

### Double-Tip-Induced Manipulation on Cu(610)

Although the relatively corrugated Cu(110) surface might provide the needed local atomic arrangements for a single rotation of the molecule, the details of the surface geometry of Cu(110) are not suited for repeated rotations. A geometrically fine-tuned corrugated surface that can serve as a proper substrate for an uninterrupted molecular rotation can be regularly stepped surfaces. We, therefore, explored the geometric structures of various stepped surfaces of Cu and found one of the most geometrically suitable vicinal surfaces of (610). This is a regularly stepped surface of the flat surface of Cu(100) with six atomic chains of (100) terraces separated by more reactive steps of (110) and more openly described by the Cu6(100) × (110) representation (see Figures S1b and 2a). The terraces on the surface are wide enough to hold the molecule between the steps and small enough to have a pivoting point at the steps so that the molecule can rotate.

**Figure 2:**
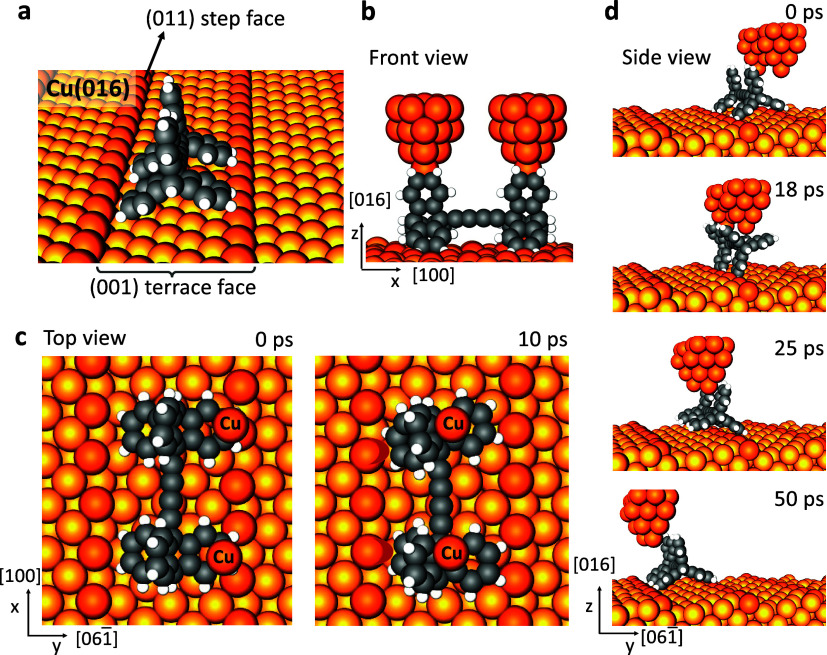
(a) Perspective view of the triptycene-based molecular wheels on the Cu6(100) × (110) stepped surface at 0 K when there is no metal tip. (b) Front view of the configuration of the molecule and the two Cu tips with a tip height of 4.6 Å. (c) Left panel pictures the initial configuration of the molecule and the double-tip at 300 K while the right panel shows the most crucial step in the rolling of the molecule on the vicinal surface of Cu(610), in which H atoms in each paddle-wheel are locked to the Cu step atoms (highlighted with red arcs in the figure). Only the single atom of the Cu-tip apex is presented to have a clear top view of the molecule. (d) Rolling of the triptycene-based molecular wheels at 300 K in the presence of double-tip (see also Movie S7). Rotation is manipulated by two separate Cu tips, and the molecule starts its rolling motion once the Cu tips move toward the molecule wheels. Snapshots show successive MD steps during a rolling process. At the intermediate step of 25 ps, the molecule is over the step, with one leg over the upper terrace and the other dangling at the lower terrace. At the final step, the molecule is positioning itself near the step on the upper terrace with the help of the double-tip.

Before performing high-temperature simulations for double tip-induced manipulations of the molecule, we calculated the activation barrier to roll the molecule on the stepped surfaces with and without the double tips: the barrier for the rolling mechanism was found to be 0.26 and 0.98 eV, respectively. Note that steps on the surface remarkably reduce the barriers compared to flat surfaces: 1.11 eV with the double tip and 2.12 eV without the double tip. These energetic values show that the stepped surfaces are a more suitable substrate for the molecule to roll on. We then executed the MD simulations to see if the molecule could be rotated around room temperature. When the molecule was first adsorbed on the Cu(610) surface, the H atoms of the wheels near the (110) step were not perfectly aligned along the step direction like a gear as in the case of the Cu(110) surface (see Figure S1a,b). However, when the two tips were positioned near the wheels, the H atoms in each paddle wheel were readily locked to the step atoms at the very beginning of the simulations (see the top view at 10 ps in Figure 2c). After the docking of the H atoms to the step atoms was completed, both wheels of the molecule were easily rotated at 300 K under the manipulation of two Cu-tip apexes (see Figure 2d and Movie S7). Remember that in our simulations on Cu(110), we did not observe any rotation of the molecule at room temperature. The full rotation of the molecule on the Cu(610) surface was also observed when the temperature was set to a lower temperature of 150 K.

### Manipulation by External Electric Field (EF) on Cu(610)

**Figure 3:**
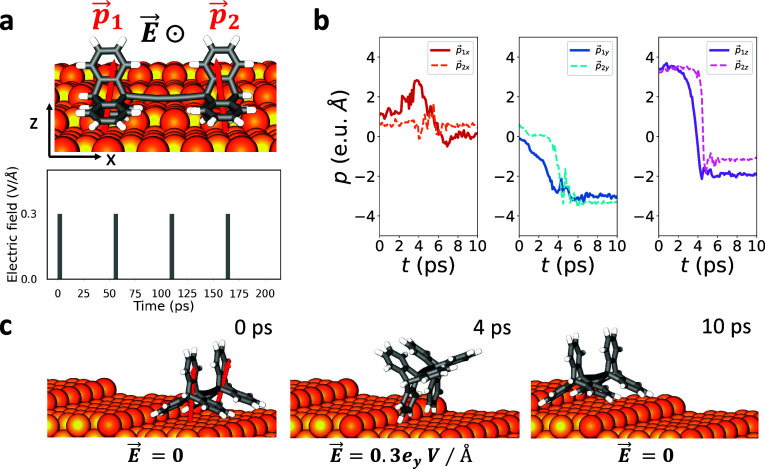
Triptycene-based molecular wheels on the Cu(610) vicinal surface. (a) Presence of electric field (EF), *E⃗*. *p⃗*_1_ and *p⃗*_2_ are the initial dipole moment vectors of left and right wheels, respectively.^27^ The bottom figure depicts the four successive switching on/off of the EF in the 216 ps simulation (see also Movie S8). *E⃗* is applied along the *y*-direction with the magnitude of 0.3 V/Å for 4 ps and then paused for 50 ps to relax the molecule after rotation. (b) Components of dipole moments during the rolling mechanism at 30 K. (c) Side views of the selected snapshots showing the rolling of the wheels in response to *E⃗*.

On the stepped surfaces, the tip-induced rolling of the molecule can be achieved only for one full rotation. Controlled application of electric field (EF), on the other hand, might provide the basic physical conditions for an uninterrupted rolling of the molecule on the surface. We, therefore, switched on EF in the MD simulations in a controlled manner and focused on the manipulation of the molecule on the Cu(610) surface by a finite tangential component of the electric field (see Figure 3). Each wheel of the molecule is a dipolar group, which provides the molecule with a strong dipole moment and thus interacts with the EF to drive the molecular rotation. Our calculations show that the dipole moments of each wheel in their initial configurations are almost perpendicular to the terrace face of the supporting stepped surface (see Figure 3a,b). When the EF is activated in the *y*-direction, the torques (τ) force the dipoles to align along the EF, and thus, the molecule is likely to rotate rather than slide once the wheels are locked to the steps which provide the pivoting points for the vertical rotation of the molecule. However, from our repeated simulations at 30 K, we conclude that constant application of the EF eventually forces the molecule to slide on the regularly stepped surface, regardless of how strong or weak the field is. On the contrary, when the EF is intermittently applied again in the *y*-direction, the uninterrupted rolling of the triptycene molecule on the Cu vicinal surface is successfully prompted. Note that to generate a torque that will force the molecule to rotate around the step, EF must be activated in the region above the central axle of the molecule, with a threshold value of 0.3 V/Å (see Figure 3a,c). Evidently, the intermittent EF allows the triptycene-based molecule to relax on the new terrace and then lock onto the consecutive step once it completes a full rotation around the previous step. Application of an EF of 0.3 V/Å for 4 ps is sufficient to rotate the molecule, and pausing the field for another 50 ps is needed to relax the molecule in its new position after the rotation. Note that the energy profiles in Figure S5 assert the time interval needed for the torque to overcome the energy barrier for the molecule to rotate. Throughout these successive switching on and off, the molecule diffused from one terrace to another via the rolling mechanism (see Movie S8). As clearly seen in the movie and in Figure 3b,c, there is a slight asynchronization in the rotations of the wheels. The initial τ⃗_1_ on the left wheel is stronger than the torque on the right wheel and thus induced the rolling earlier: τ⃗_1_ = −1.02*x̂* + 0.32*ẑ* with a magnitude of 1.07 eV and τ⃗_2_ = −0.96*x̂* + 0.17*ẑ* with a magnitude of 0.98 eV. The asynchronization in the rotation and the torque arises from the local differences in the initial dipole moments on the left and right wheels: *p⃗*_1_ = 1.08*x̂* – 0.08*ŷ* + 3.41 *ẑ* (in e.u. Å) and *p⃗*_2_ = 0.56*x̂* + 0.6*ŷ* + 3.20 *ẑ* (in e.u. Å) (see Figure 3b).

Although our calculations confirm that Cu(610) can serve as a representative vicinal surface that can facilitate the repetitive rolling of triptycene-based molecules under the influence of an external EF, the effects of terrace size on the dynamics of the rolling mechanism on these surfaces still require further investigation. To address this, we carefully conducted simulations on the same type of vicinal surfaces with shorter and larger terraces (see Table S2). Our calculations suggest that on vicinal surfaces of Cu(210) and Cu(310) with relatively shorter terraces, the molecule does not rotate but instead slides when subjected to varying strengths of an external EF. When the terrace size significantly smaller than the distance between the legs of the molecular wheel (8.20 Å), the substrate acts more like a corrugated flat surface to the molecule, which subsequently hinders the effective rotation of the molecule (see Figure S8a). In contrast, on the vicinal surface Cu(10 1 0) with relatively larger terraces compared to Cu(610) (see Table S2), the molecule rotates around the step when it is in the neighborhood. After one full rotation, a moderate EF is needed to slide the molecule near the next step on the upper terrace so that the rolling of the molecule around the new step can be initiated when a stronger EF is turned on (see Figure S9). For vicinal surface Cu(510) with terrace sizes comparable to Cu(610), the molecule repetitively rotates around the steps without sliding (see Figure S8b).

### Manipulation by External Field on Other Vicinal Surfaces

Our simulations on the vicinal surfaces with (100) terrace and (110) step orientation show that repetitive rolling of triptycene-based molecular wheels is achievable if the molecule is exposed to an intermittent EF on the terraces of an optimal size. Vicinal surfaces consist of an ordered array of steps separated by low Miller index terraces as they are crystal planes oriented a few degrees away from the low Miller index surfaces of (110), (100), and (111). Therefore, in addition to the vicinal surfaces with (100) terraces and (110) steps, other vicinal surfaces with different step and terrace orientations may also be suitable for the repetitive rolling of triptycene-based molecular wheels. Consequently, we extended our calculations to explore other potential vicinal surfaces to validate the results obtained on the Cu(610) surface. For each flat surface of (110), (100), and (111), we consider two different types of steps (see Figures S6, S7 and Table S3). Cu(540) and Cu(771) vicinal surfaces were selected to represent two types of vicinal surfaces with (110) terraces, each separated by two different set of steps. Cu(540) contains five chains of atoms on each of its (110) terraces, partitioned by periodic arrays of (100) steps. In contrast, Cu(771) accommodates four chains of atoms on the (110) terraces, separated by (111) steps (see Table S3). Although both surfaces have ideal terrace sizes that allow the molecule to roll when exposed to an external EF, in the case of Cu(771) only the wheel of the molecule, with one leg positioned in the (110) channel and locked between the front atoms of the (110) terrace chains, rotates when an EF of 0.35 V/Å is applied for 2 ps (see Figure S10a). When the EF is turned off for 50 ps to allow the molecule to relax, it becomes disoriented relative to the step, with one wheel of the molecule on the upper terrace and the other on the lower terrace (see Figure S10). For Cu(540), on the other hand, both legs rotate, but they do so asynchronously under an electric field of 0.35 V/ Å for 2 ps. When the EF is then turned off for 50 ps, the molecule adopts an orientation on the (110) terrace similar to orientation D among the four possible orientations on the (110) surface (see Figures S2 and S10b). Note that the step height of these surfaces (1.13 Å) is the lowest compared to other vicinal surfaces (see Table S3), which appears insufficient to serve as effective pivot points for rotation. Therefore, the step height is another factor that determines the type of motion of the molecule on the vicinal surfaces.

**Figure 4:**
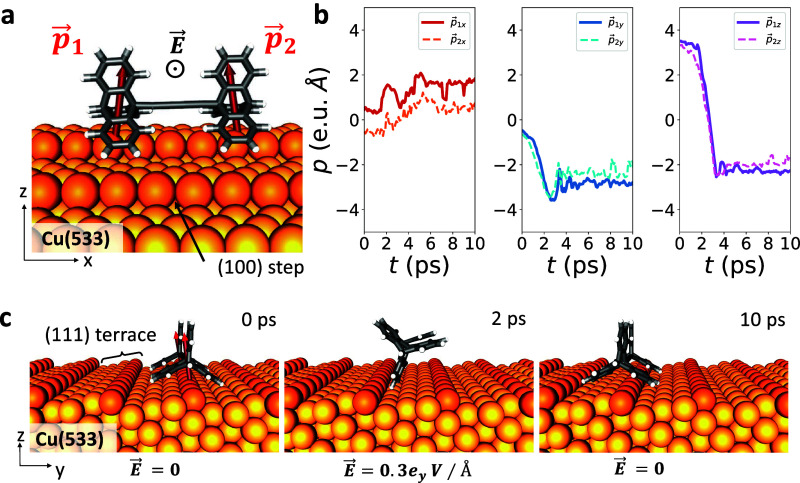
Triptycene-based molecular wheels on the Cu(533) vicinal surface. (a) Presence of electric field, *E⃗*. *p⃗*_1_ and *p⃗*_2_ are the initial dipole moment vectors of left and right wheels: *p⃗*_1_ = 0.46*x̂* – 0.46*ŷ* + 3.49*ẑ* (in e.u.Å) and *p⃗*_2_ = −0.52*x̂* – 0.65*ŷ* + 3.31*ẑ* (in e.u.Å). (b) Components of dipole moments during the rolling mechanism at 30 K. (c) Side views of the selected snapshots showing the rolling of the wheels in response to *E⃗* which is applied along the *y*-direction with a magnitude of 0.3 V/Å for 2 ps and then paused for 50 ps to relax the molecule after rotation.

The vicinal surfaces of Cu(533) and Cu(553) were examined as representatives of the vicinal surfaces with (111) terraces separated by (100) steps and (111) steps, respectively (see Figure S7c and Table S3). Since the width of the terrace on Cu(533) is less than the distance between the two legs of the molecular wheels, one leg of the wheels remains on the upper terrace whereas the other rests on the lower terrace (see Figure 4c). When the external EF is applied for 2 ps, the molecule rotates around the step. After a 50 ps relaxation period without the electric field, it returns to its original position on the new terrace, ready to rotate again. Among the vicinal surfaces considered, the synchronization of the rolling motion between the two wheels is notably optimal on Cu(533) (see Movie S9 and Figure 4). To better understand the optimal synchronization in the rotation of both wheels on the Cu(533) vicinal surfaces, we also calculated the dipole moments for each wheel during one complete rotation, as shown in Figure 4c, and plotted the results in Figure 4a,b. As seen in the plots, dipole moments of both wheels exhibit similar characteristics when the EF is turned on and off, thereby leading to similar rotational patterns for the wheels. It is worth noting that the initial direction of the torque acting on each molecular wheel is mainly along the *x*- direction (τ⃗_1_ = −1.05*x̂* + 0.14*ẑ* on the left wheel and τ⃗_2_ = −0.99*x̂* – 0.16 *ẑ* on the right wheel) with respective magnitudes of 1.06 and 1.01 eV. Furthermore, the intermittent application of the electric field within 150 ps allowed the molecule to rotate repetitively on the steps, similar to the repetitive rolling observed on Cu(610).

For Cu(553), the terrace size of 8.63 Å is larger than the separation between the legs of the wheel (8.20 Å) and can adequately hold the molecule on the terrace. However, when the system is equilibrated at 30 K, the molecule adopts a configuration in which one wheel is on the upper terrace and the other is on the lower terrace, similar to the initial configuration of the molecule on Cu(711) (see Figure S11a,b). The motion of the molecule under the influence of the electric field on Cu(553) resembles that on Cu(711), as after one complete rotation, the molecule returns to its initial orientation on the upper terrace, with one leg resting on the lower terrace (see Figure S11a,b).

Having extensively studied Cu(610), which has (100) terraces and (110) steps, we chose to focus on Cu(711) in this section as the second type of vicinal surface with (100) terraces separated by (111) steps. When the molecule initially positioned on the terrace, it adopts a configuration where one leg of the wheels is on the upper terrace and the other is on the lower, since the terrace width (7.6 Å) is shorter than the separation between the molecular legs (8.20 Å) (see Figure S11a). When the electric field is turned on, the molecule first docks at the step and then rotates. After being allowed to relax for 50 ps, it returns to its initial configuration on the new terrace, where one leg of the molecular wheels is on the upper terrace and the other is on the lower terrace. The rolling of the molecule on both Cu(553) and Cu(711) is not repetitive since additional fine-tuning of the EF is needed each time the molecule completes one rotation.

In summary, there are several factors for the effective utilization of vicinal surfaces as substrates to initiate the repetitive rolling of triptycene-based molecular wheels under the influence of an external electric field: (1) Orientation and width of terraces: Our calculations indicate that vicinal surfaces with (110) or larger terraces do not facilitate repetitive rolling. (2) Step heights: Steps with short heights cannot provide the pivot points for effective rolling. (3) Atomic arrangements along the steps: the (110) steps are optimal due to their more open atomic arrangements. Steps with (100) orientation are also favorable, as the 4-fold sites of the (100) step face provide the necessary docking sites for rolling.

## Conclusions

In conclusion, the primary factors for prompting a vertical rotation of a triptycene-based two-wheel molecule via a single/double metal tip on a metal surface are the roughness of the surface, height of the tip, and temperature. Our research has confirmed that the surface roughness of Cu(110) is effective for molecule rolling at low temperatures of 10–30 K, but not at higher temperatures. In contrast, a vicinal surface such as Cu(610) can be used as a substrate to enable the rotation of the molecule at room temperature. Our results also suggest that repetitive rolling of the molecule is achievable only when the following conditions are implemented: (1) intermittent application of EF and (2) utilization of a vicinal surface as a substrate. The crucial factors in utilizing vicinal surfaces as pivot point providers for rotation are the width and orientation of the terraces, as well as the height of the steps. For a triptycene-based two-wheel molecule, the threshold EF and the perfect substrates for continuous rolling are found to be 0.3 V/Å and the Cu(610) and the Cu(533) vicinal surfaces, respectively. We also found that the measure to estimate the strength of the field needed for the molecule to roll over the step is the energy barrier regarding the rolling mechanism for the molecule.

## Supporting Information Available

Computational cells, potential energy surface profiles, manipulations of the molecule with a tip, partial charge distributions on Cu-tip apex, potential energy profile of molecule under the electric field, and various vicinal surfaces for rolling mechanisms under the electric field (PDF)
Supplementary movie 1: rolling on Cu(110) at 10 K with the tip height of 4.6 Å (MP4)
Supplementary movie 2: rolling on Cu(110) at 30 K with the tip height of 4.6 Å (MP4)
Supplementary movie 3: sliding on Cu(110) at 80 K with the tip height of 4.4 Å (MP4)
Supplementary movie 4: sliding-rolling on Cu(110) at 80 K with the tip height of 4.6 Å (MP4)
Supplementary movie 5: rolling on Cu(110) at 30 K in the presence of double-tip with the tip height of 4.4 Å (MP4)
Supplementary movie 6: sliding on Cu(110) at 80 K in the presence of double-tip with the tip height of 4.6 Å (MP4)
Supplementary movie 7: rolling on Cu(610) at 300 K in the presence of double-tip with the tip height of 4.6 Å (MP4)
Supplementary movie 8: electric field-induced rolling of the molecule on Cu(610) at 30 K (MP4)
Supplementary movie 9: electric field-induced rolling of the molecule on Cu(533) at 30 K (MP4)
