# Supplementary material for: Repetitive Rolling of Triptycene-Based Molecules on Cu Surfaces
Source: J Am Chem Soc. 2024 Sep 19;146(39):27014–21. doi: 10.1021/jacs.4c08652 (PMC11450755; doi:10.1021/jacs.4c08652)
Supplement: Supplementary file 1 — ja4c08652_si_001.pdf [file ja4c08652_si_001.pdf]

Supporting Information: Repetitive Rolling of Triptycene-Based Molecules on Cu Surfaces

Mine Konuk,[*,†] Melihat Madran,[‡] Mehmet Tuna Uysal,[¶,||] Deniz Beşer,[¶] Alimet Sema Özen,[§,⊥] Zehra Akdeniz,[§] and Sondan Durukanoğlu[*,†]

†*Faculty of Engineering and Natural Sciences, Kadir Has University, Istanbul, 34083, Türkiye*

‡*Faculty of Engineering and Natural Sciences, Sabancı University, Istanbul, 34956, Türkiye*

¶*Robert College, Istanbul, 34345, Türkiye*

§*Faculty of Arts and Sciences, Piri Reis University, Istanbul, 34940, Türkiye*

||*Present address: Department of Electrical and Computer Engineering, Princeton University, Princeton, New Jersey 08544, USA*

⊥*Present address: Faculty of Science, Department of Chemistry, Marmara University, Istanbul, 34722, Türkiye*

E-mail: mine.konuk@khas.edu.tr; sondan@khas.edu.tr

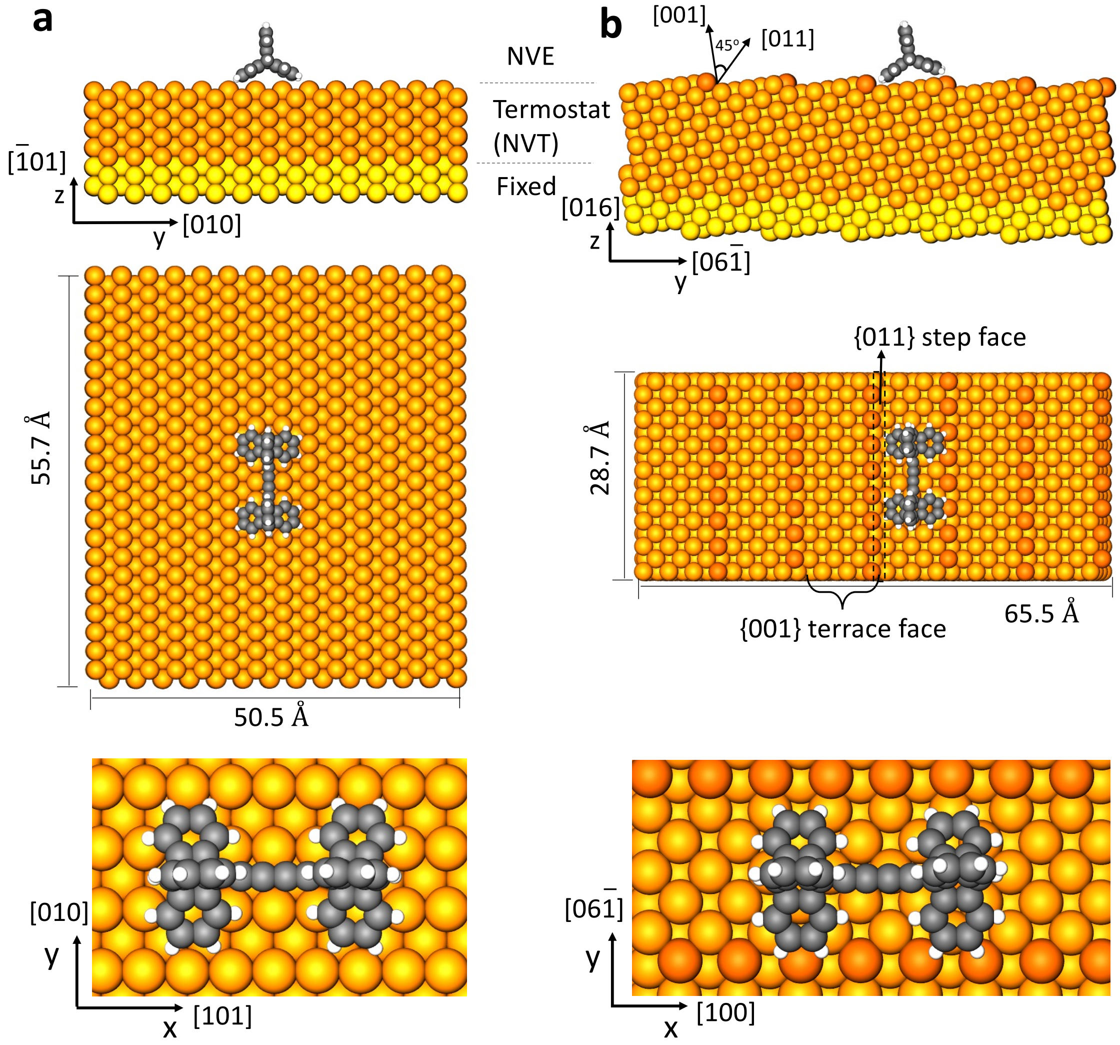

Figure S1: Computational cells for Cu(110) and Cu(610): the triptycene-based molecular wheels on the flat surface of Cu(110) (a) and on the vicinal surface of Cu(610) (b). The cell for Cu(110) contains 22×14×12 atoms along the $x-$, $y-$, and $z-$directions while that for Cu(610) consists of $8 \times 42 \times 10$ atoms in the respective directions. The atoms at the four bottom layers of each Cu slab were held fixed and excluded from the thermostat (yellow atoms in (a) and (b)). All other layers of the slab were kept under thermostat, and the molecule and Cu-tip were free within the microcanonical ensemble of NVE. Charge transfer between the atoms of the top layers of the substrate, molecule and Cu-tip were allowed whereas the atoms in the bottom layers within Cu slabs were held fixed with neutral charges. Lower panels show top views of energetically favorable configurations in the absence of the tip at 0 K.

Table S1: Computational cell details of the vicinal surfaces investigated in this study. The cells contain $n_x \times n_y \times n_z$ atoms along the $x-$, $y-$, and $z-$directions, with periodic lengths of L_x and L_y along the $x-$ and $y-$ directions, respectively. In the compact notation of $n(h_tk_tl_t)\times(h_sk_sl_s)$, n stands for the number of atomic chains on a terrace whereas $(h_tk_tl_t)$ and $(h_sk_sl_s)$ represent the Miller indices of the terrace and step faces, respectively. Periodic boundary conditions are imposed along the $x-$ and $y-$ directions, while no such constraint is applied along the z-direction. All lengths are given in Å.

Surface	Compact notation n$(h_tk_tl_t)\times(h_sk_sl_s)$	L_x	L_y	$n_x \times n_y \times n_z$
Cu(110)	$\infty(110)\times(h_sk_sl_s)$	55.7	50.5	$22 \times 14 \times 12$
Cu(540)	5(110)×(100)	28.8	46.2	$8 \times 20 \times 10$
Cu(771)	4(110)×(111)	25.5	50.7	$10 \times 16 \times 10$
Cu(210)	2(100)×(110)	28.8	32.3	$8 \times 24 \times 10$
Cu(310)	3(100)×(110)	28.8	45.7	$8 \times 32 \times 10$
Cu(510)	5(100)×(110)	28.8	36.8	$8 \times 24 \times 10$
Cu(610)	6(100)×(110)	28.8	65.5	$8 \times 42 \times 10$
Cu(10 1 0)	10(100)×(110)	28.8	72.5	$8 \times 44 \times 10$
Cu(711)	4(100)×(111)	30.63	36.45	$12 \times 16 \times 10$
Cu(533)	4(111)×(100)	30.63	33.47	$12 \times 16 \times 10$
Cu(553)	5(111)×(111)	30.63	39.21	$12 \times 20 \times 10$

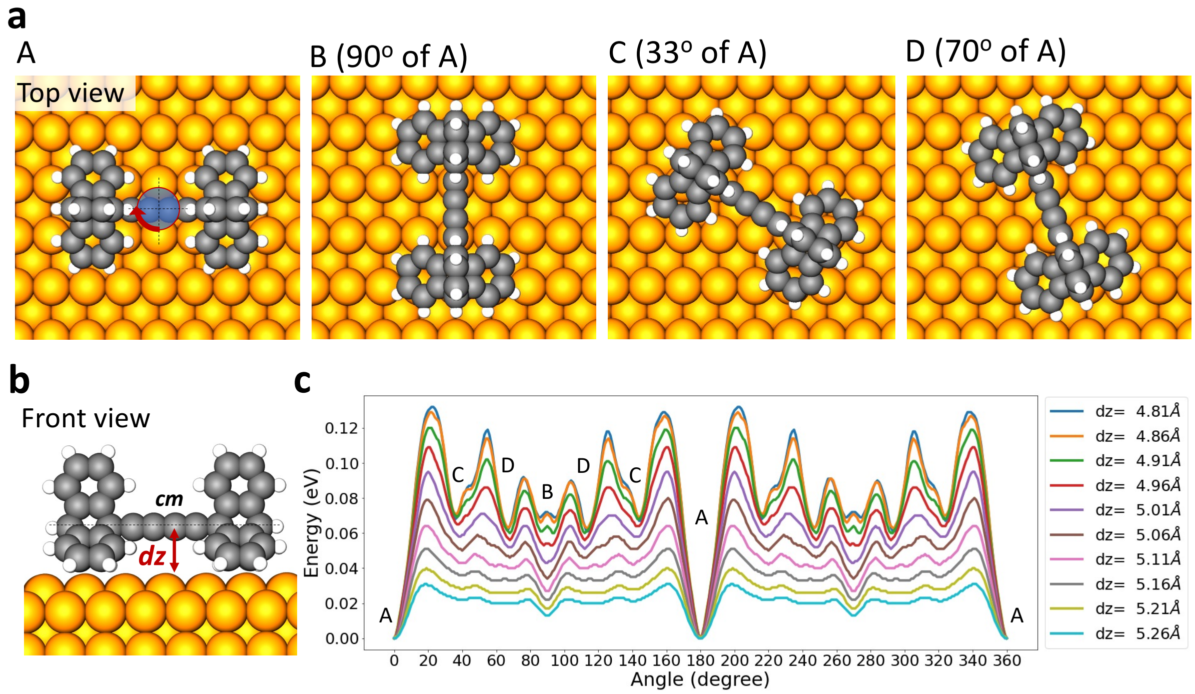

Figure S2: (a) Top views of the energetically favorable configurations of triptycene-based molecular wheels on the Cu(110) flat surface. (b) Front view of configuration A. d_z is the height of the center of mass of the molecule (cm) with respect to the surface. (c) Potential energy surface profile of the molecule when rotated around its center of mass about 360°, leading to the four preferable configurations of A, B, C, and D.

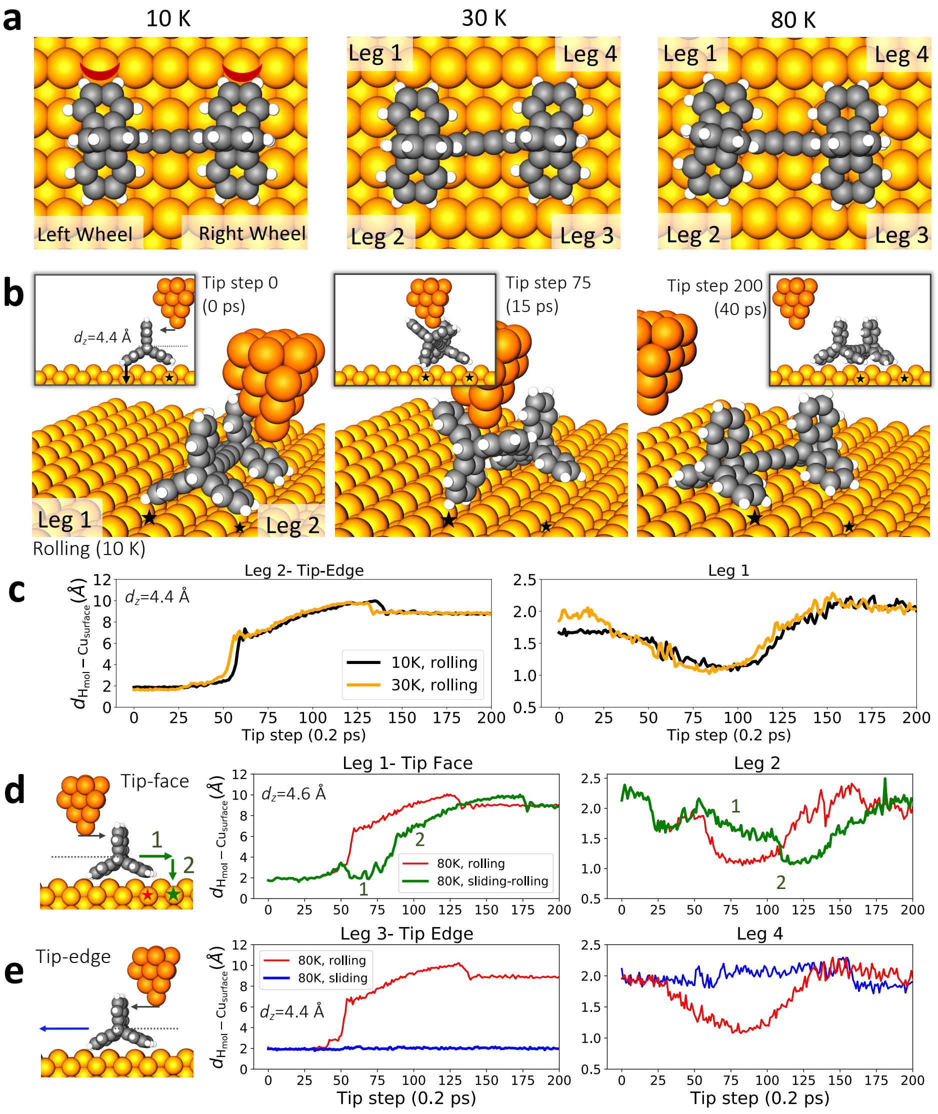

Figure S3: (a) The snapshots of initial configurations of the molecule before the manipulation with a tip at 10 K, 30 K and 80 K. In the initial configurations at 10 K and 30 K, the legs of both wheels are firmly attached to the (110) channel atoms. However, at 80 K, due to thermal effects, the legs are no longer in the docking sites that allow easier rotation. In each manipulation at different temperatures, the Cu-tip (edge or face) moves towards one wheel (left or right) of the molecule with a speed of 0.5 Å/ps. (b) Snapshots of the rolling mechanism of the left wheel of the molecule at 0 ps, 15 ps, and 40 ps while manipulated by a single edge type tip at 10 K, with a tip height of 4.4 Å. (c) The vertical separation between the hydrogen atoms of the legs of the left wheel and the Cu surface atoms during the rolling of a single wheel (the left wheel) at 10 K and 30 K when manipulated by a single edge type tip. (d)-(e) The vertical separation between the hydrogen atoms of the legs of the two wheels and the Cu surface atoms during the rolling of a single wheel (the left wheel) at 80 K when manipulated by a single edge/face type tip.

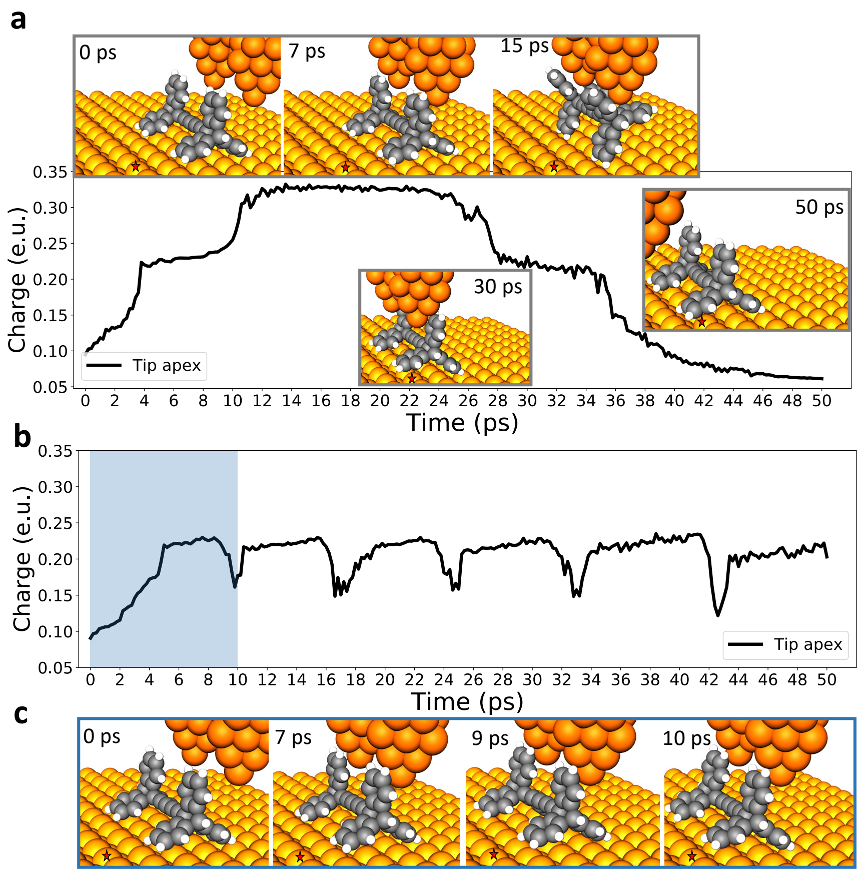

Figure S4: Variation of partial charge distribution on Cu-tip apex while pushing the molecule on Cu(110). (a) The snapshots show the rolling mechanism of the molecule when manipulated by two tips at 10 K, with a tip height of 4.4 Å. (b) Partial charge distribution on Cu tip apex during six repetitive sliding mechanisms of two wheels of the molecule at 10 K, with a tip height of 4.6 Å. (c) Snapshots of the sliding mechanism of two wheels of the molecule at 0 ps, 7 ps, 9 ps and 10 ps when manipulated by two tips at 10 K, with a tip height of 4.6 Å. Color coding of orange, dark orange, gray, and white stand for substrate Cu atoms, Cu-tip apex atoms, carbon atoms, and hydrogen atoms, respectively.

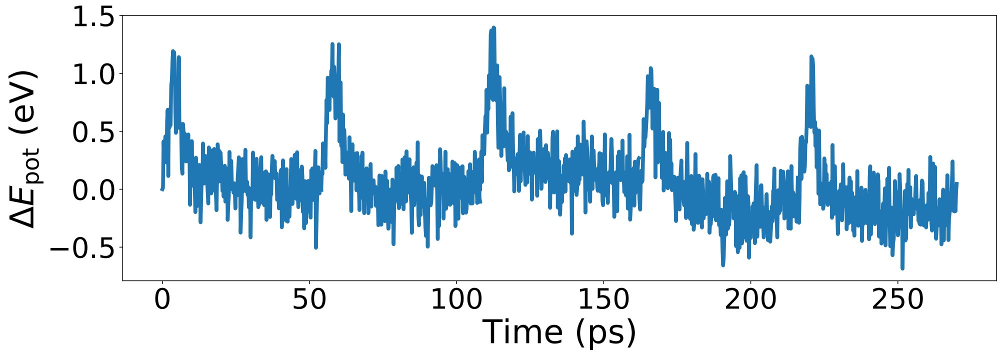

Figure S5: The change in the potential energy (ΔE_{pot}) of the molecule on Cu(610) when the external electric field (EF) is applied intermittently at 30 K.

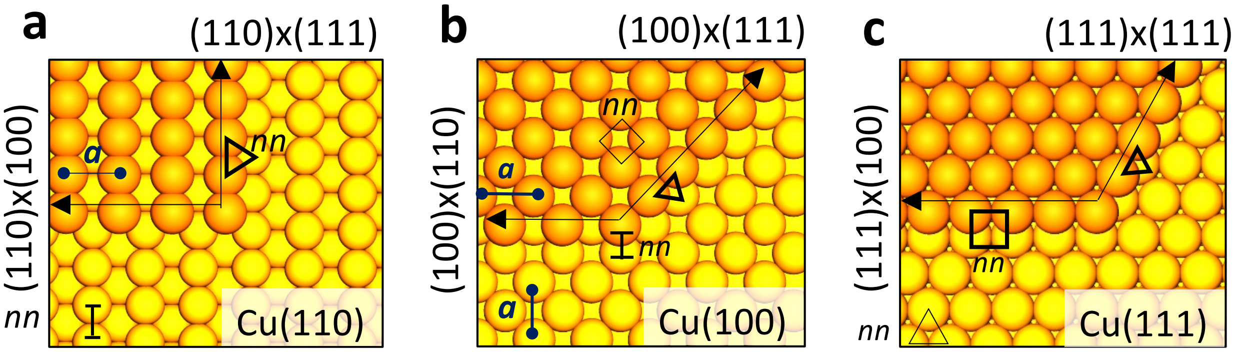

Figure S6: Top views of the two distinct step configurations chosen for this study on each low-Miller-index surface: (a) the (100) and (111) steps on the flat surface of (110), (b) the (110) and (111) steps on the flat surface of (100), and (c) the (100) and (111) steps on the flat surface of (111). Here, nn (2.55 Å) indicates the nearest neighbor distance, while a (3.61 Å) is the lattice constant of Cu. $(hkl) \times (hkl)$ Miller indices on the side and the top represent the corresponding terrace and step orientations, respectively.

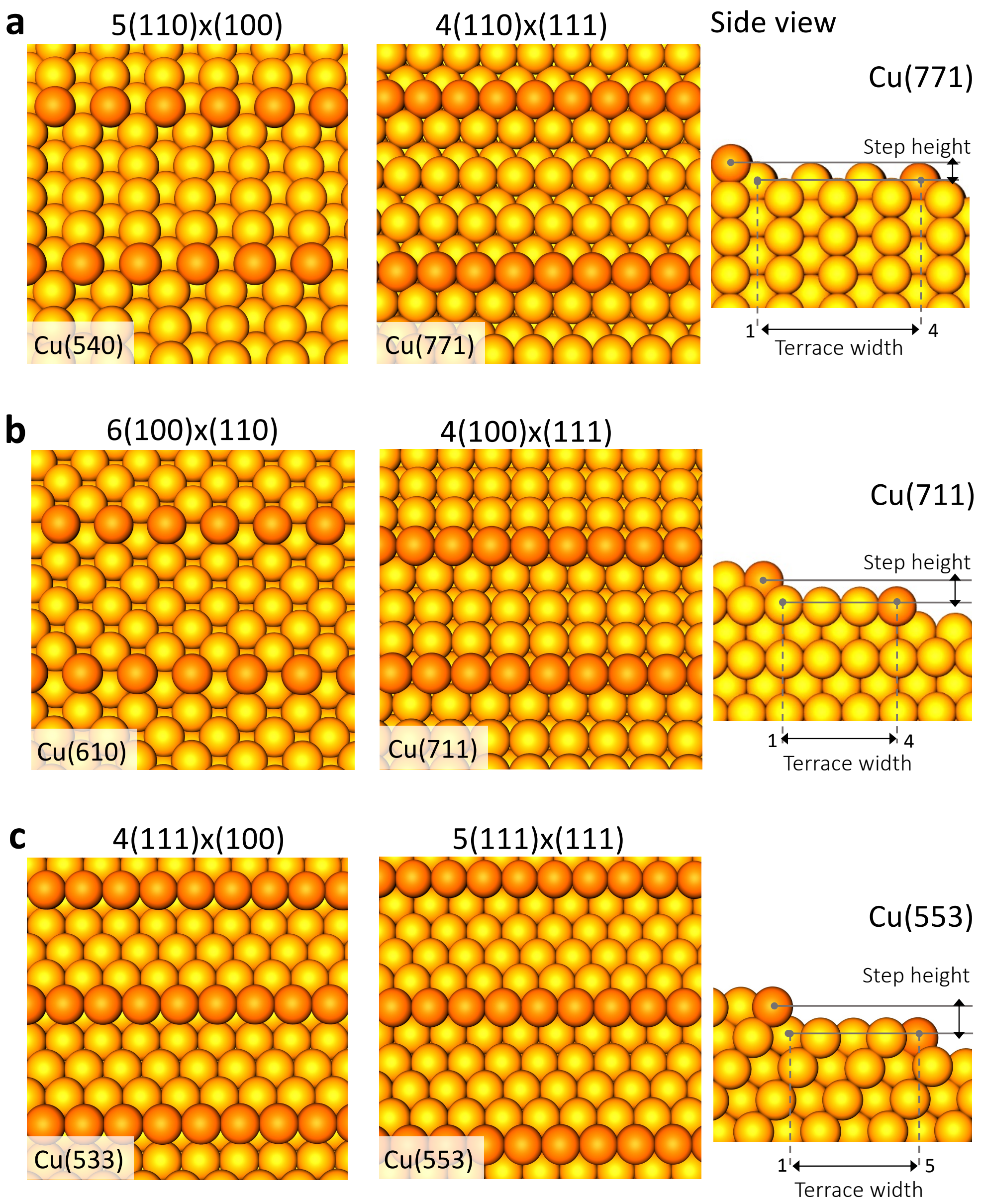

Figure S7: Top and side views of the vicinal surfaces studied in this work: (a) vicinal surfaces of Cu(540) and Cu(771) with (110) terraces separated by (100) and (111) steps, respectively, (b) vicinal surfaces of Cu(610) and Cu(711) with (100) terraces separated by periodic arrays of (110) and (111), respectively, and (c) vicinal surfaces of Cu(533) and Cu(553) with (111) terraces partitioned by (100) and (111) steps, respectively.

Table S2: Structural properties of vicinal surfaces with (100) terraces investigated in this study. In the compact notation of $n(h_t k_t l_t)\times(h_s k_s l_s)$, n stands for the number of atomic chains on a terrace whereas $(h_t k_t l_t)$ and $(h_s k_s l_s)$ represent the Miller indices of the terrace and step faces, respectively. Here, the step heights and the terrace widths are values obtained from systems equilibrated at T=0 K and given in Å.

Surface	Compact notation $n(h_t k_t l_t)\times(h_s k_s l_s)$	Step height	Terrace width
Cu(210)	2(100) × (110)	1.60	1.58
Cu(310)	3(100) × (110)	1.62	3.52
Cu(510)	5(100) × (110)	1.62	7.13
Cu(610)	6(100) × (110)	1.62	8.82
Cu(10 1 0)	10(100) × (110)	1.62	16.07

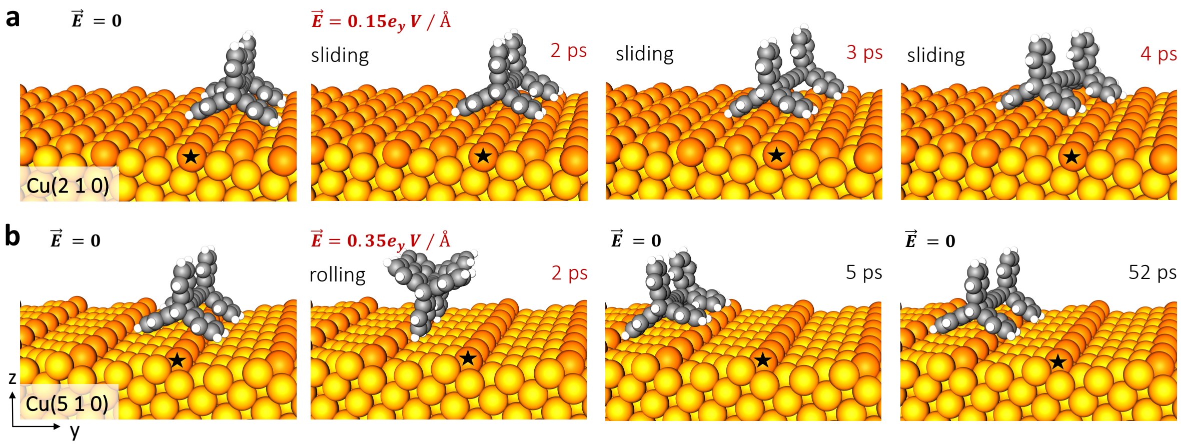

Figure S8: Motions of the triptycene-based molecular wheels at 30 K under the influence of external electric field: (a) on Cu(210) and (b) on Cu(510). The color coding indicates different atoms: orange for substrate Cu atoms, dark orange for Cu-step atoms, gray for carbon atoms, and white for hydrogen atoms.

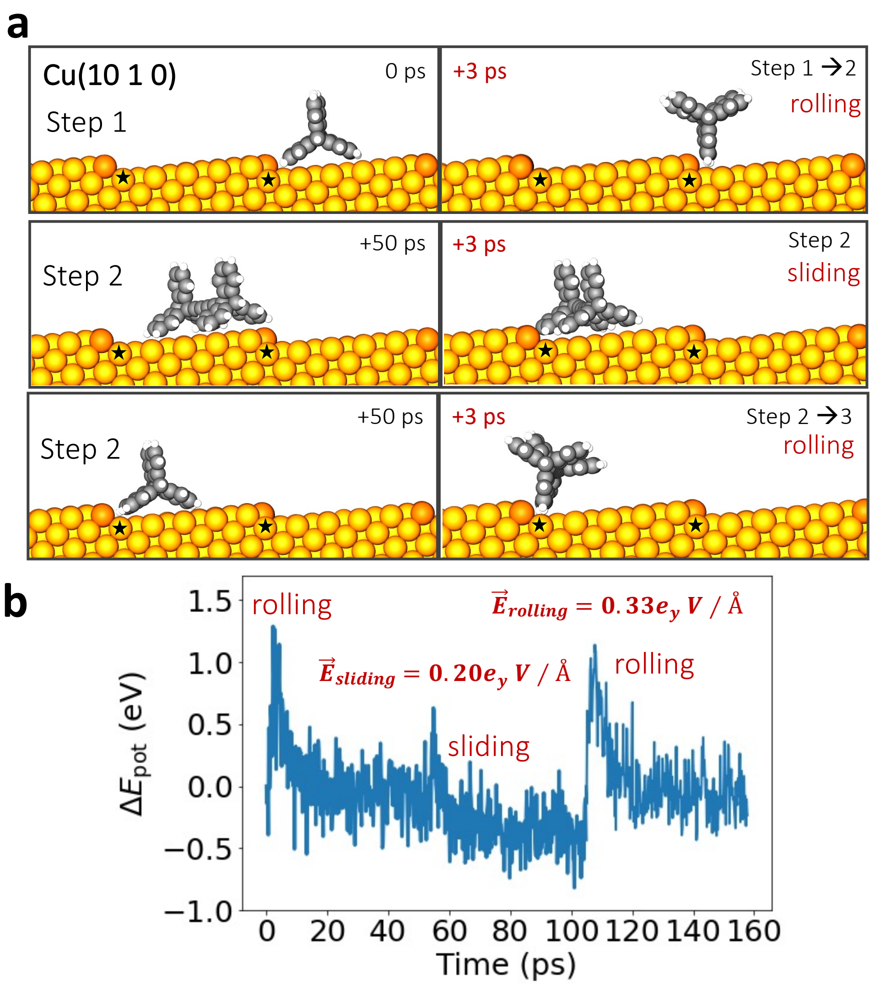

Figure S9: (a) Motion of the molecular wheels at 30 K under the influence of external electric field on Cu(10 1 0): First, the wheels rotate around the step, then slide towards the next step on the upper terrace, followed by another rotation around the step on the upper terrace. (b) Change in the potential energy of the molecule during its motion. The color coding distinguishes different atoms: orange for substrate Cu atoms, dark orange for Cu-step atoms, gray for carbon atoms, and white for hydrogen atoms.

Table S3: Structural properties of vicinal surfaces with (110), (100) and (111) terrace orientations investigated in this study (see also Figure S7). In the compact notation of $n(h_t k_t l_t)\times(h_s k_s l_s)$, n stands for the number of atomic chains on a terrace while $(h_t k_t l_t)$ and $(h_s k_s l_s)$ represent the Miller indices of the terrace and step faces, respectively. Here, the step heights and the terrace widths are values obtained from systems equilibrated at T=0 K and given in Å.

Surface	Step notation $n(h_t k_t l_t)\times(h_s k_s l_s)$	Step height	Terrace width
Cu(540)	5(110) × (100)	1.13	10.19
Cu(771)	4(110) × (111)	1.16	10.94
Cu(610)	6(100) × (110)	1.62	8.82
Cu(711)	4(100) × (111)	1.65	7.60
Cu(533)	4(111) × (100)	1.97	6.57
Cu(553)	5(111) × (111)	1.90	8.63

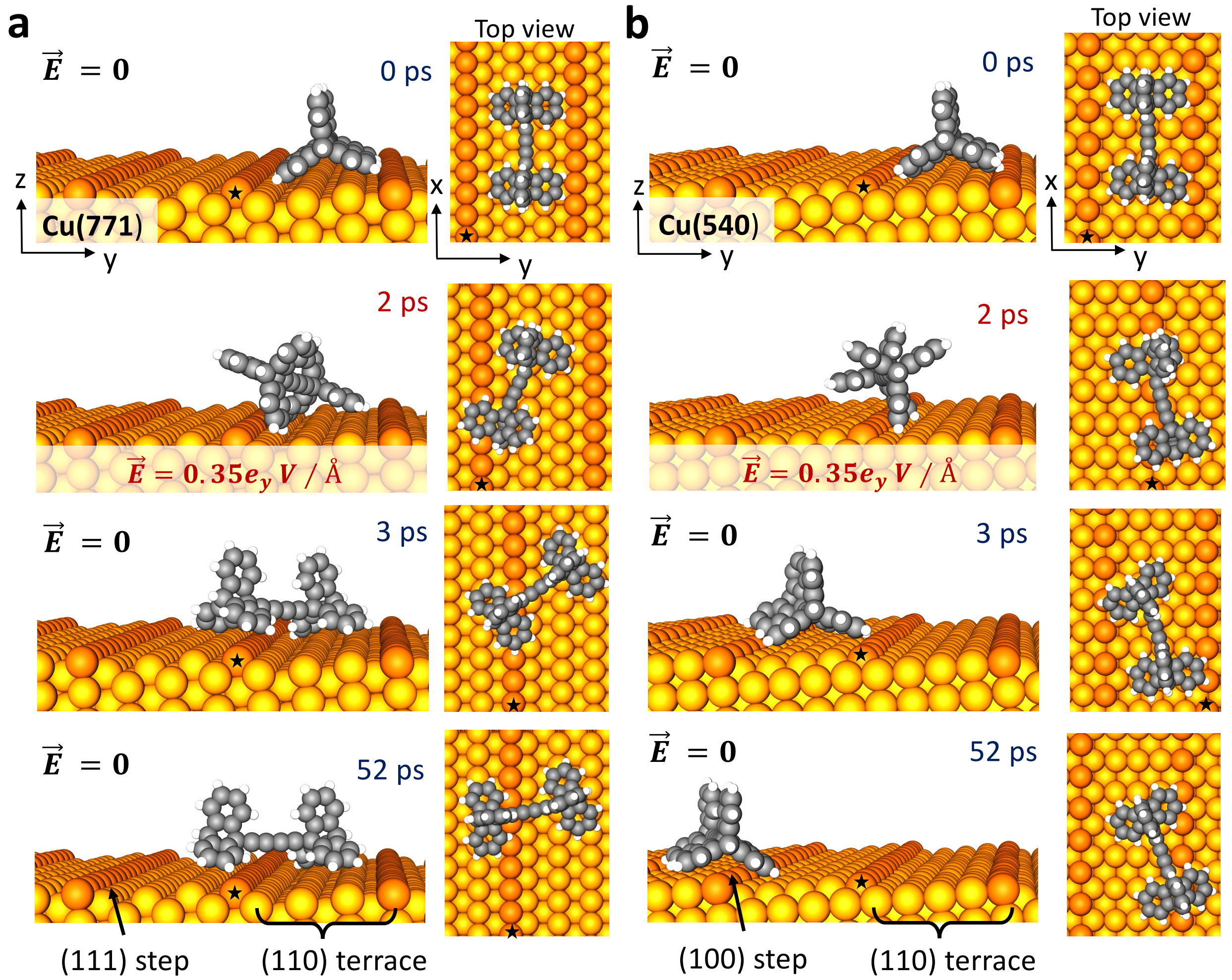

Figure S10: Motion of the molecular wheels at 30 K under the influence of intermittently applied EF: (a) on Cu(771) and (b) on Cu(540). The color coding indicates the following atomic species: orange for substrate Cu atoms, dark orange for Cu-step atoms, gray for carbon atoms, and white for hydrogen atoms.

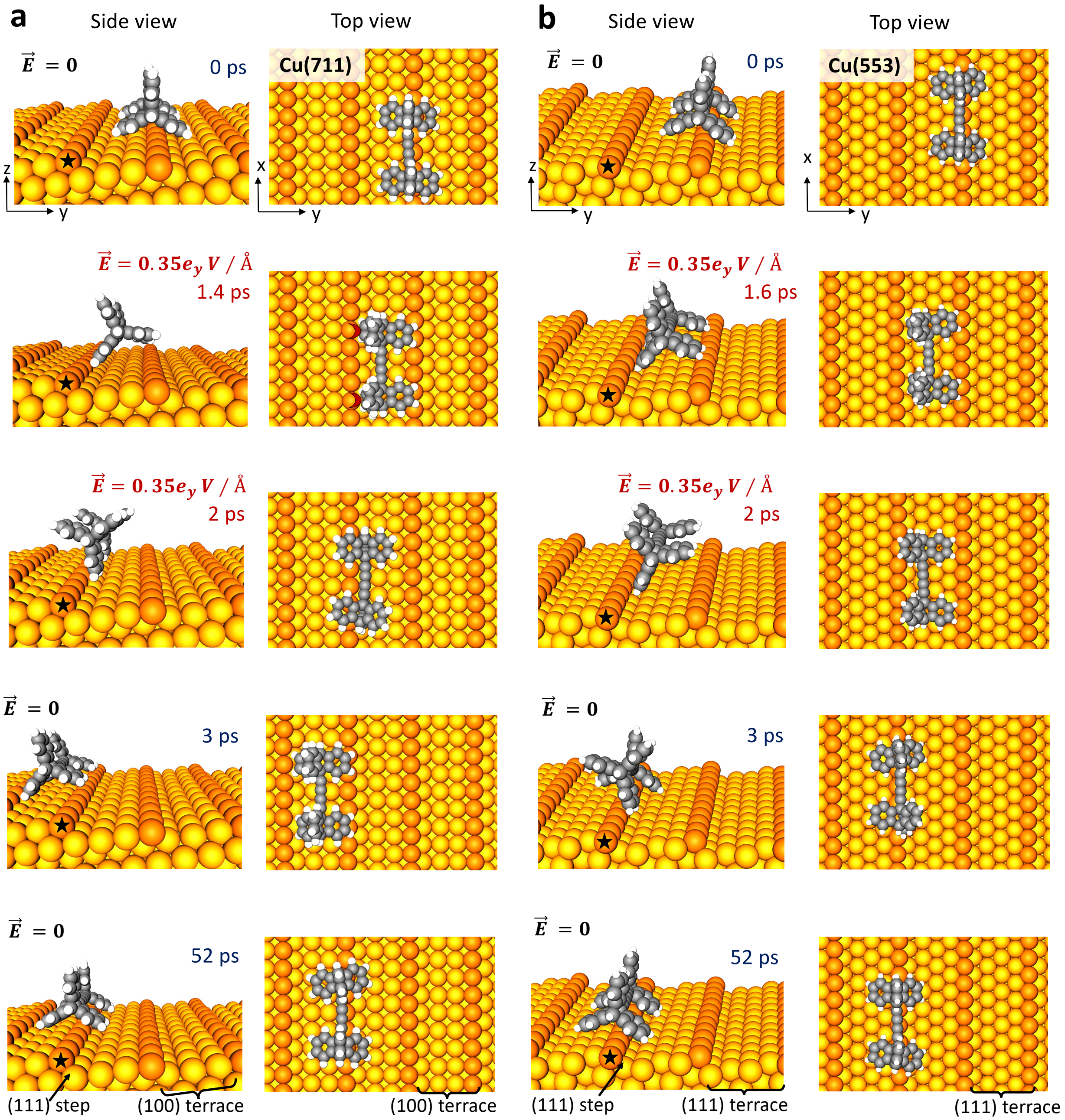

Figure S11: Motion of the triptycene-based molecular wheel at 30 K when exposed to an intermittent EF: (a) on Cu(711) and (b) on Cu(553).
